# Minimally invasive versus open radical trachelectomy for early-stage cervical cancer: protocol for a multicenter randomized controlled trial in China

**DOI:** 10.1186/s13063-020-04938-3

**Published:** 2020-12-14

**Authors:** Xiaopei Chao, Lei Li, Ming Wu, Huanwen Wu, Shuiqing Ma, Xianjie Tan, Sen Zhong, Jinghe Lang

**Affiliations:** 1 Department of Obstetrics and Gynecology, Peking Union Medical College Hospital, Shuaifuyuan No. 1, Dongcheng District, Beijing, 100730 China; 2 Department of Pathology, Peking Union Medical College Hospital, Beijing, 100730 China

**Keywords:** Cervical cancer, Radical trachelectomy, Progression-free survival, Overall survival, Clinical pregnant rate, Live birth rate, Quality of life, Randomised controlled trial

## Abstract

**Background:**

There are limited data comparing the oncologic and fertility outcomes of patients with early-stage cervical cancer (CC) treated by minimally invasive radical trachelectomy (MIRT) or abdominal radical trachelectomy (ART). The purpose of this multicenter study is to compare the oncologic and fertility outcomes of patients treated by MIRT or ART in a randomized controlled manner in China.

**Methods:**

This is a noninferiority, randomized controlled trial performed at 28 Chinese centers; the study is designed to compare the oncologic and fertility outcomes of patients treated by MIRT (robot-assisted or laparoscopic RT) or ART. Patients will be recruited if they have been diagnosed with stage IA1 (with lymphovascular space invasion), IA2, or IB1 CC (with a maximum tumor diameter ≤ 2 cm) in the FIGO 2009 staging system and histological subtypes of squamous carcinoma, adenocarcinoma, or adenosquamous carcinoma and if they are also aged 18 to 40 years. These candidates will be randomly assigned to undergo MIRT or ART. The primary endpoint will be disease-free survival. Secondary endpoints will consist of overall and disease-free survival rates, fertility outcomes, and quality of life. A total of 414 patients are needed to accomplish the study goal, with 90.1% power at a 0.050 significance level to detect an equivalence hazard ratio of 0.75 in the ART group, considering 20% loss to follow-up.

**Discussion:**

The results of the trial should provide robust evidence to surgeons regarding options for the surgical approach in patients with early-stage CC who have a strong willingness to preserve fertility.

**Trial registration:**

ClinicalTrials.gov NCT03739944. Registered on November 14, 2018

**Supplementary Information:**

The online version contains supplementary material available at 10.1186/s13063-020-04938-3.

## Background

With an estimated 570,000 new cases and 311,000 deaths in 2018 worldwide, cervical cancer (CC) ranks as the fourth most frequently diagnosed cancer and the fourth leading cause of cancer death in women [1]. The majority of CCs occur in low-income and middle-income countries. CC represents a major health challenge in China [2]. The standard surgical procedure for patients with early-stage CC is radical hysterectomy (RH) and pelvic lymphadenectomy. However, in the last two decades, with the widespread use of CC screening programs, there has been an increase in early-stage CCs diagnosed in women of childbearing age who desire childbearing. Therefore, fertility-sparing surgery (FSS), such as conization and radical trachelectomy (RT), has become one of the options for young women with early-stage CC.

Abdominal radical trachelectomy (ART) was first described by Romanian gynecologist E. Aburel in 1956 [3] and was “rediscovered” by the team of Smith and Ungár in the 1990s [4]. Vaginal RT, first performed by Daniel Dargent, involves resection of the cervix, the upper part of the vagina, and the proximal part of the parametria via a vaginal approach, combined with laparoscopic pelvic lymphadenectomy, while preserving the uterine corpus [5]. However, vaginal dissection in Dargent’s procedure must be performed by skilled surgeons. Furthermore, with the progress of minimally invasive technology, minimally invasive RT (MIRT) has allowed for a reduced length of hospital stay and estimated blood loss; decreased analgesic requirements, blood transfusion rates, and complication rates; earlier recovery of physiological functions; and improved esthetic outcomes [6].

A recent multicenter randomized controlled trial (RCT) [7] and a large cohort study [8] of minimally invasive versus abdominal RH for CC showed that the minimally invasive surgery (MIS) resulted in decreased survival compared with the laparotomic approach. The results of both studies aroused great controversy over the surgical approaches of CC worldwide. However, few data have been reported to evaluate the oncologic and/or fertility outcomes of RT with different surgical approaches, except for retrospective studies and systematic reviews. In a previously published paper from our center, ART versus vaginal RT yielded recurrence rates of 0 and 9.8% [9]. In a systematic review of stage IA to IIA CC, the recurrence rates after ART, laparoscopic RT, and robot-assisted RT were 4.7% (31/660), 6.3% (15/238), and 2.2% (2/89), respectively [10]. In another systematic review, among patients with abdominal, vaginal, laparoscopic, and robotic RT, the incidences of cervical stenosis were 11.0%, 8.1%, 9.3%, and 0%, respectively [11]. The lack of a prospective study promoted the conception of our RCT.

## Methods

### Design

**Fig. 1:**
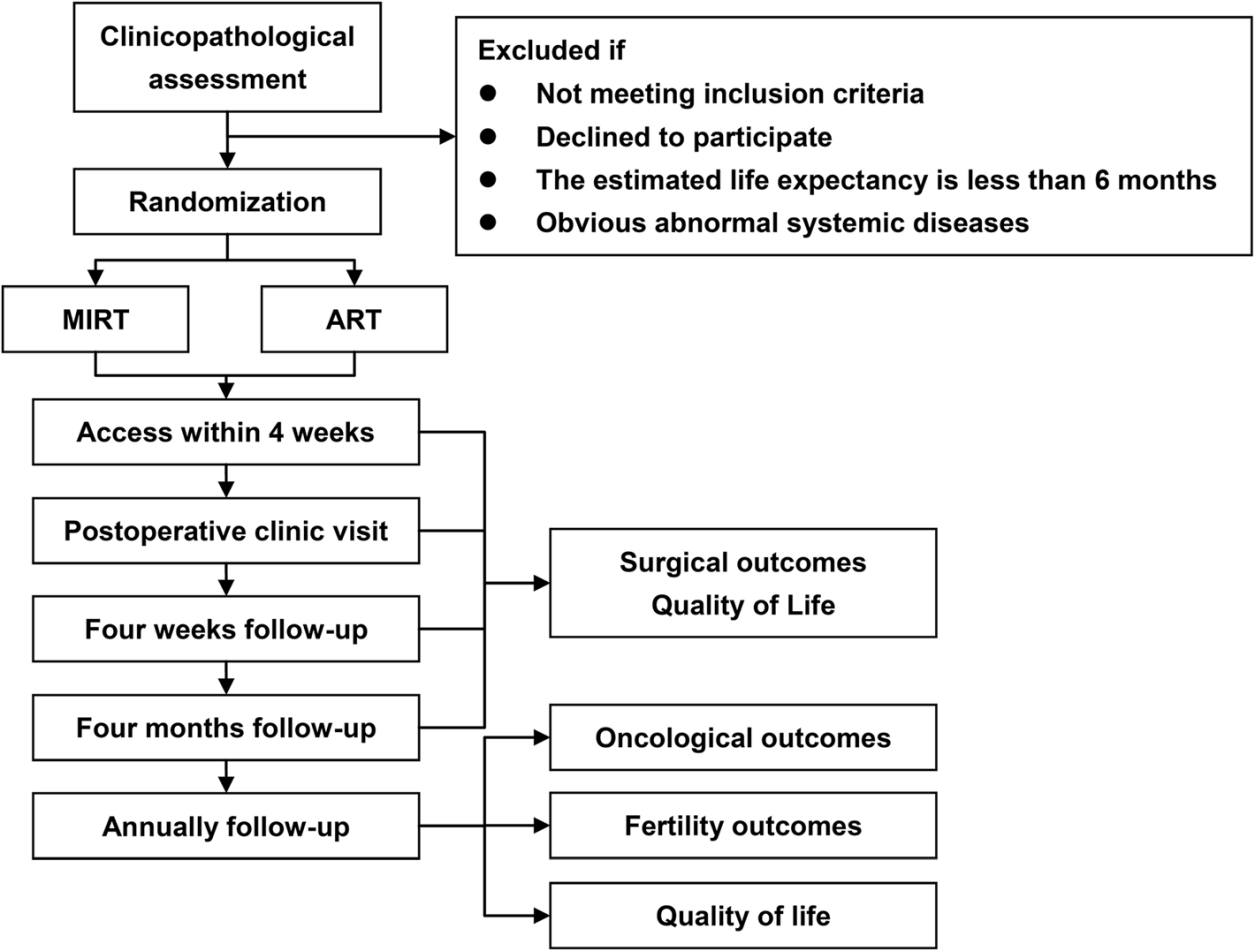
CONSORT flow diagram of the study. ARH, abdominal radical hysterectomy; MIRT, minimally invasive radical trachelectomy; ART, abdominal radical trachelectomy

**Fig. 2:**
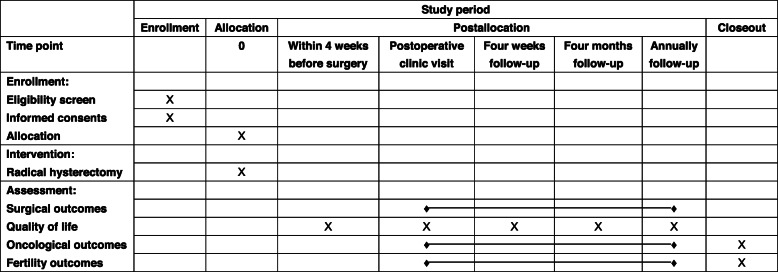
SPIRIT figure of the study

The present investigation is a randomized, controlled, noninferiority, two-arm trial comparing the efficacy of different surgical approaches of RT on the oncology and fertility outcomes in Chinese patients with early-stage CC (Figs. 1 and 2). A total of 28 domestic centers will recruit 414 patients (207 per group) from December 31, 2018, to December 31, 2020. All the surgeries are to be performed by the specialists designated in the research center (the primary investigators are listed in Supplementary Table 1). The patients are randomized on the day of written consent obtainment by the random data website (http://random.your-data.cn:8095/random), which assigns each patient a randomization number. Specified surgeons from all centers are in charge of enrolling participants. When one potential eligible patient is enrolled, her information will be sent to Dr. LL, who will generate the allocation sequence and assign participants to interventions. The allocation, together with treatment information, will be discussed with the patient by her surgeons when patients attend the clinic to get results, and relevant informed consent will be signed. On the consent form, participants will be asked if they agree to the use of their data should they choose to withdraw from the trial. Participants will also be asked for permission for the research team to share relevant data with people from the universities taking part in the research or from regulatory authorities, where relevant. This trial does not involve collecting biological specimens for storage. Model consent form and other related documentation given to participants and authorized surrogates are available from the corresponding author on request.

Figure 1 refers to the CONSORT flow diagram. Survival outcomes (disease-free survival [DFS] and overall survival [OS]) will be analyzed comprehensively, as well as the fertility and pregnancy outcomes and quality of life (QoL).

Any important protocol modifications, happened before or during the trial, will receive a deliberative evaluation by the primary investigator and be approved by the plenary session consisting of all participating centers. Once the modifications were approved, a copy will be sent to the funders and all centers. The clinical trial registry will be updated due to the modifications. Any deviations from the protocol will be fully documented using a breach report form.

#### Inclusion criteria

Inclusion criteria are as follows: patients with International Federation of Gynecology and Obstetrics (FIGO) 2009 stage IA1 with lymphovascular space invasion (LVSI), IA2, or IB1 with tumor size ≤ 2 cm CC; no evidence of upper endocervical involvement (tumor confined to the cervix as proved by preoperative imaging, and a cranial extent of the tumor that is at least 1 cm away from the internal os); no lymph node spread or distant metastasis; histological subtype of squamous cell carcinoma, adenocarcinoma, or adenosquamous carcinoma; aged 18 to 40 years; ability to provide fully informed written consent for participation in this study; strong desire to preserve fertility and no history of fertility impairment; ability to undergo long-term follow-up after surgery; and Eastern Cooperative Oncology Group (ECOG) performance status [12] of 0 to 1.

#### Exclusion criteria

The exclusion criteria were as follows: histological subtype of neuroendocrine, clear cell, and serous cell type or metastatic carcinoma; clinically advanced disease (IB1 with tumor size > 2 cm or stages IB2 to IV); current pregnancy; age younger than 18 or older than 40 years; having received neoadjuvant chemotherapy or radiotherapy; without a systematic preoperative imaging evaluation (pelvic MRI with or without PET/CT); unwillingness to participate in this study; and preexisting malignancy, severe systemic disease or severe mental illness.

### Main objective and primary endpoint

The primary objective of the analysis is to evaluate the 5-year DFS related to different surgical approaches for radical trachelectomy (RT). The primary endpoint will be recurrence confirmed by imaging or histology.

### Secondary objectives and secondary endpoints

The secondary objectives are as follows:

1. To evaluate the OS at the 5-year follow-up
2. To assess different strategies of RT in terms of fertility and obstetric outcomes
3. To compare the incidence of perioperative complications of different surgical approaches
4. To compare major perioperative complications within 28 days after surgery and major postoperative complications
5. To determine the general and specific QoL

Secondary endpoints will therefore be the following:

1. All-cause mortality
2. Fertility outcomes consisting of cervical incompetence, natural pregnancy, and infertility treatment such as intrauterine insemination or IVF-ET; obstetric outcomes consisting of the time interval between the RT surgery and pregnancy; the overall mean length of time during which conception was attempted; the number of pregnancies; the mode of delivery; and the outcome of each pregnancy, such as spontaneous abortion, induced abortion, drug abortion, preterm birth, premature rupture of membranes, fetal growth restriction, and full-term birth
3. Perioperative complications consisting of injury of the ureter, bladder, bowel, blood vessels, or nerves, and vaginal bleeding, and postoperative complications consisting of postoperative pelvic infection, pelvic lymphocele, intrauterine infection, Asherman syndrome, abnormal menstruation, urinary tract infection, hydronephrosis, intestinal obstruction, chronic pain, varices at the site of uterovaginal anastomosis, and fistula formation
4. Major perioperative and postoperative complications consisting of early postoperative complications (cerebrovascular, pulmonary, and renal diseases; ileus; abdominal wound complications; septicemia; thromboembolism; and lymphocele formation and secondary infection) and delayed postoperative complications (lymphedema, vaginal evisceration)
5. QoL assessment comprising the authorized Chinese edition of EORTC QLQ-C30 [13] and its CC module QLQ-CX24 [14], the 19-item Female Sexual Function Index (FSFI) [15], and the PFIQ-7 questionnaire [16].

### Description of the parameters for assessing efficacy endpoints

The primary endpoint is a composite endpoint, which will be collected at each visit. Surgical complications will be assessed by surgeons and/or a specialist if he or she has been consulted for a complication. Obviously, a double-blinded trial was not feasible, i.e., the surgeons and clinicians managing the patients have to explain the operation method and risks to the patients, and in turn, the patients must sign their informed consent. However, the information of patients and surgeons are blinded to performers dealing with data analysis.

### Learning curve

In this study, all surgeons have been qualified by the existing continuous clinical data and survival outcomes of at least 10 patients with early-stage CC who have undergone RT, including at least 5 cases of MIRT. Meanwhile, the surgeons should provide all surgical reports and at least two integrated copies of surgical videos as the competence of these surgeries. These data and videos will be presented as supplementary material for the final report. The survival outcomes are used to assess the learning curves of participating surgeons, and the videos are used to assess the surgical skills.

### Surgical treatment

Patients with early-stage CC who are eligible for enrollment will be randomly assigned to the MIRT group or ART group. Evaluation of the pelvic nodes in patients with early-stage CC will be performed. The pelvic lymph nodes may be assessed with a complete lymphadenectomy or via sentinel lymph node mapping. Surgical details, including uterine artery preservation, the cerclage material and position of knot placement, and the method to prevent cervical stenosis, will be recorded in the surgical records.

### ART

Entry into the abdomen may be accomplished via a midline vertical incision to gain adequate access to the pelvis. After excising the pelvic lymph nodes, the paravesical and pararectal spaces are developed. The round ligaments are grasped with Kelly clamps for uterine manipulation, and the vesicocervical space is developed to dissect the bladder off the cervix. Care is taken to preserve the ovary pelvic funnel ligaments and utero-ovarian vessels, as these are the main blood supply to the remaining uterus. The uterine vessels are preserved, while their descending branches are resected at the level of the isthmus. Once complete ureterolysis is performed to the tunnel of Wertheim, the uterine vessels are divided at their origin from the hypogastric vessels. The parametria and paracolpos are mobilized with the trachelectomy specimen. The posterior cul-de-sac is incised, and the uterosacral ligaments are divided at their origin. The vagina is then incised to perform an anterior colpotomy 1–2 cm distal to the external cervical os. This incision is then carried circumferentially until the specimen is completely separated from the vagina. The lower uterine segment is estimated, and clamps are placed at the level of the internal os. The cervix is then incised with a knife approximately 5 mm below the internal os. Patency of the remaining cervical canal may be achieved with the use of a Foley or Malecot catheter followed by the placement of an endocervical cerclage. The lower uterine segment is then sutured to the vaginal mucosa via interrupted or continuous sutures [17].

### MIRT

The procedure with either a laparoscopic or robotic approach is the same with regard to the abdominal approach. A uterine manipulator must be avoided. A vaginal excision must be made under vaginal exposure rather than under peritoneal exposure.

### Safety and adverse events

The adverse events and their severity will be judged by the criteria of the Common Terminology Criteria for Adverse Events [18]. This trial will be conducted in compliance with this study protocol.

### Data management

All medical information required according to the protocol will be collected via the case report forms based on the website, facilitating the real-time, central assessment of the data completeness and patient follow-up. This information will include demographic data, the surgical approach, the skin-to-skin operative time, EBL, and the surgical energy devices. The pathogenic material will also be collected, including the results regarding the tumor histology subtype, FIGO stage, grade (well, moderately or poorly differentiated), tumor size, depth of stromal invasion, LVSI, lymph node status, and surgical margin status. After RT, data on surgical complications, bladder/intestinal function recovery, QoL, fertility and obstetrics outcomes, and disease recurrence and survival information will also be followed.

The questionnaires will be completed, the ovarian reserve function will be evaluated, and a urodynamic study (UDS) and anorectal manometry will be assessed within 4 weeks before surgery, at 4 months postoperatively, at 12 months postoperatively, and then once a year thereafter. After the RT (Table 1), the patients will be required to attend the customed follow-up regularly, at 3- to 6-month intervals in the first 2 years after completing the treatment, 6- to 12-month intervals from the third to the fifth year after treatment, and then at 1-year intervals thereafter. The effects of the follow-up scheme (pelvic examination, the ThinPrep cytological test and/or human papillomavirus testing, transvaginal ultrasonography, an abdominal ultrasound scan, chest radiography, and serum tumor marker, i.e., squamous cell carcinoma and Ca125) will be evaluated at each visit.

**Table 1:** Lists of clinical assessments at different time points

Check-up time	Inspection items	Documentors	Directors
Within 4 weeks before RT	Urodynamic assessment	CRF	Research centers
	Anorectal manometry	CRF	
	QoL assessment	CRF	
	Sexual function scale	CRF	
	Ovarian reserve function	CRF	
Ten to 14 days after RT	Operation details	CRF	Research centers
	Residual urine	CRF	
	Perioperative complications	CRF	
Four weeks after RT	Postoperative complications	CRF	
	Pathological report	CRF	
Four months after RT	Urodynamic assessment	CRF	Research centers
	Anorectal manometry	CRF	
	QoL assessment	CRF	
	Ovarian reserve function	CRF	
	Postoperative adjuvant therapy	CRF	
	Data curation	Database	Main center
Every 12 months after RT	Urodynamic assessment	CRF	Research centers
	Anorectal manometry	CRF	
	QoL assessment	CRF	
	Sexual function scale	CRF	
	Ovarian reserve function	CRF	
	Pregnant desires and outcomes	CRF	
	Survival outcome	CRF	
	Data curation	Database	Main center
	Follow-up time, scheme, and results	Database	

*CRF* case report form, *QoL* quality of life, *RT* radical trachelectomy

This trial will be conducted in compliance with the current version of the protocol, with all statutory and regulatory requirements. The Data Safety Monitoring Committee (Supplementary Table 2) will regularly receive study data, including complications, patient conditions, and survival outcomes. This committee will be composed of physicians, administration staff, and scientific researchers, who are not participating in the study as investigators and who will be provided with available collected data during the study. The committee reserves the right to terminate the study at any time for medical or administrative reasons. In the case of loss to follow-up, the surgeons or investigators will do their best to contact each patient involved to identify if he/she is alive. If an individual leaves the research study prematurely, data related to the participant can still be used unless an objection was recorded when the patient signed the consent form. If consent is withdrawn, no data about the individual may be used unless the patient states in writing that he/she does not object.

For participants who consent to participate the trial but deviate from intervention protocols, i.e., refuse the randomized allocation, the surgical and follow-up data will still be collected the same as participants following the protocol, which will be analyzed according to intention to treat.

The trial results will be communicated to the funders, investigators, and other relevant groups via publications, reporting results in databases, data sharing arrangements, and social media (WeChat).

### Description of statistical methods

#### Sample size calculation

Systematic reviews have revealed that the rate of disease recurrence after ART is 3.8 to 4.7% [10, 19]. The 5-year DFS of ART in a Chinese study was 96.5% [20]. In our study, we assume that the recurrent rate of ART of 5 years is 5%. A noninferiority log-rank test with an overall sample size of 331 subjects (165 in the reference group and 166 in the treatment group) achieves 90.1% power at a 0.050 significance level to detect an equivalence hazard ratio of 0.75 when the actual hazard ratio is an equivalence hazard ratio of 1.00, and recurrence rates of 5 years in the MIRT and ART group are 7 and 5% [21]. Considering the possible 20% rate of loss to follow-up, 414 patients are needed to accomplish the study goal.

#### Statistics

Continuous variables conformed to the normal distribution will be described with means and standard deviations, and discrete variables not conformed to the normal distribution will be summarized with medians, ranges, and interquartile ranges. The *t* test will be used for continuous variables, and the chi-square test or Fisher’s exact test will be used for categorical variables. To evaluate the strength of associations, bivariate and multivariable logistic regression analyses will be used, and the strength of associations will be expressed as hazard ratios (HR) with 95% confidence intervals (95% CI). Kaplan-Meier plots will be generated for recurrence and death rates between the groups, and the log-rank test will be applied for the eventual significant differences. The survival outcomes will be compared according to intention to treat and per protocol. The analysis of intention to treat is available for protocol nonadherence. These statistical analyses will be performed using SPSS 22.0 (SPSS Inc., Chicago, IL, USA). All analyses will be two-sided, and significance will be set at a *P* value of 0.05.

### Strength and limitations of this study

- ► To our knowledge, this is the first multicenter RCT designed for the comparison of oncologic and fertility outcomes of different surgical routes of RT, despite a registered retrospective multicenter trial [22].
- ► With the advantage of sufficient patients with early-stage CC in the study centers, this study can be implemented as soon as possible.
- ► This study takes the surgeon as one of the important parameters and as the main consideration, which significantly differs from the LACC trial [7]. For the first time, the learning curve for MIS will be taken into the survival analysis.
- ► This study will make uniform reports of the pathological outcomes. The comprehensive and meticulous pathological data will support the information about the prognosis.
- ► The emphasis on the individual surgeon’s experience and skill will most likely limit generalization.
- ► This study will not evaluate the type of suture material used to perform the cerclage, such as braided and nonbraided sutures, or the position of the knot placement and the time of cerclage removal before delivery.
- ► This RCT will explore the effect of different methods to prevent cervical stenosis, such as a Nelaton catheter, an intrauterine device, and a Smit sleeve, as well as the time of removal of these materials.

## Discussion

In our study, we have enrolled women younger than 40 years, with stage IA1 disease with LVSI, stage IA2, and smaller stage IB1 tumors (≤ 2 cm diameter); without evidence of lymph node metastases on imaging; with evidence of endocervical extension on MRI; and with a desire to preserve fertility. At present, five different FSS procedures are available for patients with CC: conization, a simple trachelectomy, VRT, MIRT (including laparoscopic and robot-assisted routes), ART, extraperitoneal RT (a novel fertility-preserving option) [23], and neoadjuvant chemotherapy (NACT) followed by FSS (conization/simple trachelectomy or RT). MIS has gained wide acceptance secondary to the implied advantages of a faster return to normal activity and diet, reduced hospital stay and postoperative discomfort, and decreased scarring and rate of adhesive. Although the open approach is more radical in terms of parametrial and paracervical resection [10], it has theoretical drawbacks, particularly the formation of adhesions and scar tissue that may ultimately impact a woman’s fertility, increased blood loss, and a prolonged hospital stay.

Additionally, with the advent of the sentinel lymph node procedure, the morbidity in this usually young patient population has continued to improve. There are adequate data to prove that oncologic outcomes, concerning recurrence and mortality, are comparable to those of RH and that the obstetrical outcomes are favorable [17]. However, a recent study has put forward contrasting conclusions; thus, we aim to evaluate the impact of different surgical approaches of RT on survival and obstetric outcomes.

One of the crucial designs is the consideration of the learning curves of surgeons. A learning curve is defined by the number of surgical procedures performed by a surgeon before he or she reaches an accepted plateau in objective outcomes such as operative time, estimated blood loss (EBL), complication rate, and surgical performance [24]. Research defining learning curves in gynecologic oncology surgery is limited but has shown that gynecologic oncology surgeons can become proficient after 20 cases of robotic hysterectomy with pelvic-aortic lymph node dissection and that their performance can be continually improved in the period between 50 and 70 cases [25]. Compared with vaginal RT, ART has a shorter learning curve, requires minimal additional training, and has a higher oncological radicality. ART is similar to the abdominal RH procedure that most gynecologic oncologists are familiar with [26–28]. This suggests that experience is critical in laparoscopic and robotic procedures [29]. However, there is little literature about the learning curve for MIRT. Therefore, each study center would appoint only one specific expert who will manage all the major procedures of RT, and their quality would be evaluated by the amount and survival outcomes of the surgeries.

### Trial status

Recruitment of participants started in December 2018, and the last participant is expected to reach the primary endpoint (5-year follow-up) in December 2025. Primary data analysis will begin in December 2023. The naturalistic follow-up phase of the trial will continue until December 2025. The protocol version number and date were 3.0 and December 23, 2018, respectively.

## Supplementary Information

**Additional file 1: Supplementary Table 1.** Study centers and their primary investigators, contact staff and phone numbers.

**Additional file 2: Supplementary Table 2.** Members of the Data Safety Monitoring Committee.

## Data Availability

The datasets generated and/or analyzed during the current study are available in the public repository, http://117.50.88.118, and are also available from the corresponding author on reasonable request.

## Abbreviations

MIRT: Minimally invasive radical trachelectomy
ART: Abdominal radical trachelectomy
CC: Cervical cancer
FSS: Fertility-sparing surgery
RT: Radical trachelectomy
MIS: Minimally invasive surgery
RCT: Randomized controlled trial
DFS: Disease-free survival
OS: Overall survival
LVSI: Lymphovascular space invasion
QoL: Quality of life
NSRT: Nerve-sparing radical trachelectomy
EBL: Estimated blood loss
LQ: Leiden Questionnaire
UDS: Urodynamic study
PVR: Postvoid residual volume
MFR: Maximum flow rate
AFR: Average flow rate
MUCP: Maximal urethral closure pressure
FSFI: Female Sexual Function Index
UIQ-7: Urinary Impact Questionnaire
CRAIQ-7: Colorectal-Anal Impact Questionnaire
POPIQ-7: Pelvic Organ Prolapse Impact Questionnaire
HR: Hazard ratios
CTA: Computed tomography angiography
NACT: Neoadjuvant chemotherapy
