# Supplementary material for: Minimally invasive versus open radical trachelectomy for early-stage cervical cancer: protocol for a multicenter randomized controlled trial in China
Source: Trials. 2020 Dec 14;21:1022. doi: 10.1186/s13063-020-04938-3 (PMC7734762; doi:10.1186/s13063-020-04938-3)
Supplement: Supplementary file 1 — Additional file 1: Supplementary Table 1. Study centers and their primary investigators, contact staff and phone numbers. [file 13063_2020_4938_MOESM1_ESM.docx]

Supplement Table 1

Study centers and their primary investigators, contact staff and phone numbers.

| **Study centers** | **Serial numbers** | **Primary investigators** | **Contact staffs** | **Phone numbers** |
| --- | --- | --- | --- | --- |
| Anhui Provincial Cancer Hospital | 01 | Weidong Zhao | Yan Jiang | +86-133-5551-3031 |
| Beijing Cancer Hospital | 02 | Yunong Gao | Qian Li | +86-136-9305-6667 |
| Cangzhou Central Hospital | 03 | Shikai Liu | Hairong Yao | +86-182-3373-9102 |
| Dalian Maternity Hospital affiliated to Dalian Medical University | 04 | Jiyong Jiang | Xiuying Wang | +86-180-9885-7006 |
| Zhongda Hospital Southeast University | 05 | Yunlang Cai | Qinfen Zhang | +86-137-7066-9549 |
| Gansu Provincial Hospital | 06 | Hailin Wang | Bo Qu | +86-139-1903-2731 |
| First Affiliated Hospital of Gannan | 07 | Xiaoying Xie | Jieli Zhou | +86-159-7971-1375 |
| Guangdong Maternal and Child Health Hospital | 08 | Xiping Luo | Bin Wen | +86-135-8031-3014 |
| Cancer Hospital Affiliated to Guangxi Medical University | 09 | Zhijun Yang | Bingbing Zhao | +86-139-0786-0220 |
| Harbin Medical University Cancer Hospital | 10 | Ge Lou | Bairong Xia | +86-186-0451-6165 |
| Fourth Hospital of Hebei Medical University/Cancer Hospital of Hebei Province | 11 | Shan Kang | Min Feng | +86-152-0001-3026 |
| Cancer Hospital affiliated to Zhengzhou University | 12 | Li Wang | Hongmin Chen | +86-136-1371-8339 |
| The Affiliated Cancer Hospital of Xiangya School of Medicine, Central South University | 13 | Yile Chen | Yanxiang Tang | +86-137-2388-2463 |
| Union Hospital, Tongji Medical College, Huazhong University of Science and Technology | 14 | Zehua Wang | Shaohai Wang | +86-136-0715-0901 |
| Jilin Cancer Hospital | 15 | Chunying Gao | Jinxia Pu | +86-135-7894-5886 |
| Xijing Hospital, Air Force Military Medical University | 16 | Biliang Chen | Jia Li | +86-139-9190-7656 |
| Liaoning Cancer Hospital & Institute | 17 | Danbo Wang | Zaiqiu Long | +86-189-0091-8582 |
| Shandong Cancer Hospital | 18 | Naifu Liu | Depu Zhang | +86-188-8831-3632 |
| Shaanxi Province People’s Hospital | 19 | Lihong Chen | Fen Li | +86-152-2933-4369 |
| Renji Hospital, Shagnhai Jiaotong University School of Medical | 20 | Kaijiang Liu | Qing Liu | +86-183-2150-1410 |
| Shenyang Women’s and Children’s Hospital | 21 | Yulin Shi | Yansong Liu | +86-139-4000-7179 |
| West China Second University Hospital, Sichuan University | 22 | Ping Wang | Zhengyu Li | +86-189-8215-1025 |
| Tianjin Central Hospital of Gynecology Obstetrics | 23 | Pengpeng Qu | Jianguo Zhao | +86-138-2109-2762 |
| Chinese PLA General Hospital | 24 | Yuanguang Meng | Wen Yang | +86-138-1043-2069 |
| Peking Union Medical College Hospital | 25 | Ming Wu | Lei Li | +86-139-1198-8831 |
| Cancer Hospital Chinese Academy of Medical Sciences | 26 | Bin Li | Yating Wang | +86-188-0019-7622 |
| Chongqing University Cancer Hospital/Chongqing Cancer Hospital | 27 | Qi Zhou | Jing Wang | +86-135-9431-8836 |
| Sun Yat-sen University Cancer Center | 28 | Jundong Li | Fan Yang | +86-185-6510-1099 |
