# Supplementary material for: Minimally invasive versus open radical trachelectomy for early-stage cervical cancer: protocol for a multicenter randomized controlled trial in China
Source: Trials. 2020 Dec 14;21:1022. doi: 10.1186/s13063-020-04938-3 (PMC7734762; doi:10.1186/s13063-020-04938-3)
Supplement: Supplementary file 2 — Additional file 2: Supplementary Table 2. Members of the Data Safety Monitoring Committee. [file 13063_2020_4938_MOESM2_ESM.docx]

Supplement Table 2

Members of the Data Safety Monitoring Committee.

| **Role** | **Name** | **Affiliations** | **Duties and research directions** |
| --- | --- | --- | --- |
| Chairman | Aijun Sun | Peking Union Medical College Hospital | Reproductive Medicine and Real World Clinical Research Specialists |
| Committee member | Hui Huang | Peking Union Medical College Hospital | Director of Scientific Research, Executive and Epidemiologist |
| Committee member | Jiaxin Yang | Peking Union Medical College Hospital | Gynecologic oncologist |
| Committee member | Fengzhi Feng | Peking Union Medical College Hospital | Gynecologic oncologist |
| Committee member | Lihong Zhu | Beijing Obstetrics and Gynecology Hospital, Capital Medical University | Director of Radiotherapy Department, Oncology Radiotherapy Specialist |
| Committee member | Yingyi Wang | Peking Union Medical College Hospital | Oncologist |
| Committee member | Peng Peng | Peking Union Medical College Hospital | Assistant, Gynecologic Oncology Center, Gynecologic Oncology Specialist |
| Committee member | Jiansheng Wang | Environment and Health Data Center | Director of Center, Epidemiologist, and Expert of Big Data System Research |
| Committee member | Ling Lu | Peking Union Medical College Hospital | Head nurse of gynecological oncology, nursing specialty |
| Committee member | Xiaojuan Tian | Peking Union Medical College Hospital | Head nurse of gynecological oncology, nursing specialty |
| Committee member | Mei Mei | Institute of Medical Information/Medical Library | Expert in Large Data System Research |
| Committee member | Bin Liu | Albo (Shanghai) Medical Devices Co., Ltd. | Medical administration |
| Committee member | Huan Li | Albo (Shanghai) Medical Devices Co., Ltd. | Biomedical and Epidemiologist |
| Committee member | Peng Wang | Beijing Kewei Yongdao Trading Co., Ltd. | Medical administration |
